# Caries of Human Teeth

**Published:** 1883-04

**Authors:** Frank Abbott

**Affiliations:** New York


ARTICLE II.

### REMARKS MADE BEFORE THE BROOKLYN DENTAL SOCIETY, MONDAY EVENING, FEBRUARY 12, 1883, UPON "CARIES OF HUMAN TEETH."

BY FRANK ABBOTT, M. D., OF NEW YORK.

*Mr. President and Gentlemen of the Brooklyn Dental Society:*

In discussing this subject, I want it distinctly understood that I do it with no feeling except that of a desire to get at the truth. If any gentleman, no matter who he may be, nor what view he may take of this subject, will convince me that I am wrong, and that he is right, I will at once abandon my position, and as heartily advocate his as I now do my own. It seems, however, that I am to-night placed in rather an awkward position. The subject for discussion, as stated by yourself, Mr. President, and printed upon the notices for this meeting, is in the form of a question, as follows: "Etiology of Dental Caries, Acids or Germs! Which!" Now, without taking a second thought, I should say neither; but that second thought tells me that the beginning of the carious process in all teeth is effected by means of acids, produced by the decomposition of food, saliva, etc., which lodge around and between, as well as in depressions, or upon irregular surfaces of the teeth.

Perhaps before proceeding to consider this condition of pathology, it may be well for us all to understand alike a tooth in its physiological state. Chemically, the enamel of a tooth is said to be composed of about three and a half parts in a hundred of organic matter, and the balance ninety-six and a half parts inorganic, the dentine of about twenty-eight parts organic, and seventy-two inorganic in a hundred, and the cement about thirty-three organic and sixty-seven inorganic. I give this analysis, not that I think it absolutely correct for all teeth, as I have no doubt a different result would be obtained from the chemical analysis of the teeth of almost any two persons you might select; and I have no doubt, further, that considerable water would be found in every instance, reducing perhaps both the organic and inorganic portions, as we now understand it. But what I wish you to understand is the fact that a tooth is an organ, as perfectly and beautifully formed as the eye, or any other of the delicately constituted organs of the human body; that it contains as much organic material in proportion to its inorganic as is consistent with the strength and resisting power demanded of it; and that a portion only of that organic material is possessed of life.

Now, with this understanding of the tooth in a physiological condition, let us examine it pathologically as to the process of caries, and try and understand if possible its causes and progress. Before, however, I give you my views, or rather reiterate my views (as they have changed but very little since they were given to the Dental Profession in 1879), I will take the liberty of quoting from other gentlemen who have investigated and written upon this subject since that time. In the January number of the Dental Cosmos appears an article by Dr. W. D. Miller of Berlin, which was read before the "American Dental Society of Europe," at Ostend, in the course of which article he says : "A mixture of 68.0 grams saliva x 1.0 bread, 0.5 meat x 0.5 sugar, kept for forty-eight hours at the temperature of the human body, generated more than sufficient acid to decalcify the entire crown of a molar tooth." * * * * * *

"There remains still to be answered the questions : Do bacteria ever penetrate directly into perfectly sound enamel or dentine, and do they perform any part in the decalcification ?

"I have already referred to the gradual diminution of the bacteria in number as we go from the outer to the inner margin of the preparation, (i. e., from the surface to the deeper parts of the dentine), till at the inner border but few or none of the tubuli are found to be infected. This fact, which leads us to the conclusion that the microorganisms cannot penetrate beyond that point to which the tissue has been softened by the action of acids, may be readily confirmed by the examination of the softened tissue taken from different depths of a cavity of a carious tooth. * *

"The microscopic examination showed the decayed part to be filled with bacilli and micrococci, but in not a single case have I found them to pass beyond the softened (carious) into the sound dentine." This, mind you, in sections of dentine so thin that they were readily examined with a power of from 1,000 to 1,500 diameters."

In "teeth of poor structure" (poorly calcified) are found numerous irregular microscopic cavities (interglobular spaces). These cavities frequently communicate with one another, and, through cracks or fissures, with the surface of the tooth, in which case they may become filled with micrococci. The latter are completely confined within the cavities, and do not penetrate the normal dentine." * *

"Pieces of perfectly sound dentine, handled with great care, so as to be kept as free as possible from all foreign matter, were placed in small vials and covered with a drop of distilled water. These were then infected with leptothrix, bacilli and micrococci from decayed tooth-bone, and kept at a temperature of from 35 to 38 C. If, now, the organisms were capable of decalcifying the tooth substance, we should expect: 1. A softening of the tooth-bone. 2. The infection of the softened part. 3. An increase in the number of bacteria and cloudiness of the liquid; and 4, Since the bacteria could accomplish the decalcification only through the generation of an acid, we would expect an acid reaction of the liquid."

"These flasks we observed for four months. For the first few days an increase in the number of bacteria was apparent, but as soon as all matter upon the surface of the pieces and at the exposed ends of the entinal fibrils was consumed, the numbers diminished, and at the end of the four months only now and then a micrococus was to be seen. A cloudiness of the liquid did not occur, an acid reaction could not be detected, nor were the pieces of dentine changed, either microscopically or macroscopically."

These experiments were repeated by Dr. Miller, with the addition of a greater quantity of decaying dentine, and the result was the same; not a bacteria to be found in or upon the pieces of dentine at the end of four months.

In the "general results" which he has arrived at he says: "The invasion of the fungi is always preceded by the extraction of the lime salts."

"The fungi have not the power either to penetrate or to decalcify sound dentine." Now, Mr. President and gentlemen, this is the same position taken by myself in the paper I read before the New York Odontological Society, and published in the Dental Cosmos in the February, March and April numbers, 1879. I quote:

"The indifferent elements originating through the carious process from enamel, dentine and cement, do not proceed in new formation of living matter, but become disintegrated and transformed into a mass crowded with micrococci and leptothrix. Micrococci and leptothrix by no means produce caries; they do not penetrate the cavities in the basis-substance of the tissues of the tooth, but appear only as secondary formations, owing to the decay of the medullary elements."

If these experiments have been performed in the careful manner described by Dr. Miller (and I hardly think any one will question it), and such conclusions arrived at, it would certainly seem that we must look for some element aside from micro-organisms for the destruction of teeth by the carious process.

Now, Mr. President, I propose to present to you another side of the question :

In the January number, 1883, of the "New England Journal of Dentistry," is an article by Prof. Charles Mayr, A. M., of Springfield, Mass., detailing some chemical experiments which he has made for the purpose of establishing the theory that the carious process in human teeth was the dissolving of the tooth structure by some acid, or acids, or otherwise.

The following are the results of some of his experiments :

"No acids or soluble lime-salts are in the innermost decayed mass; hence no acetic, tartaric or lactic acid had dissolved much of the lime-salts, because the acetates, lactates, etc., would not have been washed out completely from the decayed mass, but a small amount would still remain, which, being soluble, would be easily shown by oxalate of ammonia acid."

"One large decay was sliced up into several parts, and the slices analyzed according to the above plan (details of plan all given in the article, to which I would particularly call your attention, as it is worth careful perusal) :

"First slice—Outermost, very gelatinous, soft layer.

Water, 58 per cent.; organic, 26 per cent.; lime-salts, 16 per cent.

Or, omitting the water : Organic, 61 per cent.; inorganic, 39 per cent.

Second slice—Middle, water not determined, because no longer reliable. (The specimen had become a little dry.)

Organic, 55.8 per cent.; inorganic, lime-salts, 44.2 per cent.

Third slice—Innermost, white, friable mass just close to the healthy dentine; scraped out with a soft iron wire, and very crumbling.

Organic, 32.1 per cent.; lime-salts, 67.9 per cent.

This decay which was analyzed in these slices shows, therefore, from the outside, a uniform advancement to the normal composition of the tooth. It shows that the lime-salts are removed, but not in any way which the acid theory demands. * * The tooth is disorganized, the soft, friable white decay is no longer organized, though chemically differing only slightly from the tooth-substance."

Now, Mr. President, if these experiments were conducted by Prof. Mayr with the single idea to get at the truth, and from his well-known ability and standing as a chemist, I hardly think any one will doubt that, then I would respectfully ask in what position the "acid theory" of caries is left? It would seem that, to every fair, unprejudiced mind, it must share the same fate that the "septic theory" has at the hands of Dr. Miller. With these two thories so effectually disposed of, I will now present my "theory." The more I study this subject, the more I become convinced that the first lesion under all circumstances is due to the action of an acid, which, in a merely chemical way, dissolves out the lime-salts from the enamel. "The *exact acid* might be very difficult to determine, for the reason that so many kinds of food are taken into the mouth which are acid themselves (such as the different kinds of pickles, fruit, &c., &c.), or, by fermentation, readily produce acids. This acid need not be strong, for as you are well aware, it is in many cases years and years held in contact with the same spot upon the tooth. Like "the constant dropping of water that wears the stone," the constant application of a very dilute acid will eventually dissolve the lime-salts from the most perfectly calcified enamel of a tooth. Perhaps the sour decomposition is assisted locally by the action of micrococci and leptothrix, although these organisms are known to prosper only in alkaline, and not in acid fluids. These vegetable organisms are present in innumerable quantities on the healthiest gum; tartar is crowded with them; and even in the highest degree of development of tartar *caries* is *absent.* In fact, when deayed cavities in teeth become filled with tartar, the carious process is as effectually stopped as it is possible for it to be, when such cavities are *filled* in the most perfect manner, with gold or any other favorite material." (Dental Cosmos, Feb., 1879).

In dissolving the lime-salts on the surface of the enamel, (or in any depression or irregular surface of a tooth where food lodges until decomposition occurs), the living matter in that tissue becomes exposed, and at once, under the effect of the acid, becomes more or less irritated. This irritation extends into the substance of the enamel beyond the point at which absolute destruction of the tissue has taken place, (which fact may readily be determined by diligent examination of specimens of caricus enamel carefully prepared). Under this irritation, constantly applied, inflammation soon follows; this causes more or less of a swelling of the living matter, which effects the dislodgment of the lime-salts—a melting down of the glue-giving basis-substance, and a bringing to view, under a power of from 1,000 to 1,500 diameters, the medullary or embryonal elements of the enamel. As the caries reaches the dentine, the same inflammatory reaction, with the swelling of the living matter, the enlargement of the canaliculi, dislodgment of the lime-salts, and melting down of the glue-giving basis-substance, becomes more intense, just in proportion as there is more organic and living matter in the dentine than in the enamel. These lime-salts are not necessarily dissolved and taken away, but may, and I have no doubt, as Prof. Mayr's experiments show, do remain mixed with this disorganized tooth-substance.

Upon examination of a specimen of acute caries, cross-section, at a considerable distance from the disintegrated granular mass (always to be found upon the surface of caries), may be seen enlarged canaliculi, some double, some treble, some four, six, eight, yes, even fifty times as large as normal. In many instances, near the surface, as many as six or eight may be seen to have joined together. The lime-salts between and around the canaliculi having been dislodged, the glue-giving basis-substance melted down, and forming partly nodulated protoplasmic bodies, in which the living matter is brought to view, in the shape of nuclei, with occasional threads running from one protoplasmic body to another. It is this living matter, so brought to view, which in my judgment has been mistaken by some observers for organisms. Many such enlarged canaliculi remain in a tooth after the supposed carious portion has been removed and the tooth filled. In such cases the acid irritant formerly in the cavity is removed, the inflammatory condition subsides, and the lime-salts become re-deposited. In other words these deep-seated lesions heal and become as solid and to all appearances as healthy tooth-bone as ever. Could this occur, gentlemen, if these enlarged canaliculi were filled, as some observers claim, with organisms of decomposition? It certainly looks to me a little unreasonable, to say the least.

Before closing, Mr. President, I would like to ask a few questions of those who hold to the "septic theory."

1st.—Why is it that the teeth of all persons do not decay the same?

2d.—Why is it that in ninety-nine cases out of a hundred the lower front teeth (on which may be found the greatest number of organisms of any in the mouth) do not decay, while all others in the mouth do?

3d.—Why is it that teeth with the greatest amount of lime-salts, consequently the smallest amount of organic substance (that upon which, it is claimed, the organisms subsist) do not decay sooner and more rapidly than the reverse?

4th.—Why is it that a pulp canal which has held a dead and putrifying pulp for many years, upon being opened is found to be as solid and free from decay as it was before the pulp died?

With these questions, Mr. President and gentlemen, I leave the subject for those who feel disposed to answer.

